# Knowledge Graph for Breast Cancer Prevention and Treatment: Literature-Based Data Analysis Study

**DOI:** 10.2196/52210

**Published:** 2024-02-22

**Authors:** Shuyan Jin, Haobin Liang, Wenxia Zhang, Huan Li

**Affiliations:** 1 Health Department, Shenzhen Maternity and Child Healthcare Hospital, Shenzhen, China; 2 School of Economics and Statistics, Guangzhou University, Guangzhou, China

**Keywords:** knowledge graph, breast cancer, treatment, prevention, adverse reaction

## Abstract

**Background:**

The incidence of breast cancer has remained high and continues to rise since the 21st century. Consequently, there has been a significant increase in research efforts focused on breast cancer prevention and treatment. Despite the extensive body of literature available on this subject, systematic integration is lacking. To address this issue, knowledge graphs have emerged as a valuable tool. By harnessing their powerful knowledge integration capabilities, knowledge graphs offer a comprehensive and structured approach to understanding breast cancer prevention and treatment.

**Objective:**

We aim to integrate literature data on breast cancer treatment and prevention, build a knowledge graph, and provide support for clinical decision-making.

**Methods:**

We used Medical Subject Headings terms to search for clinical trial literature on breast cancer prevention and treatment published on PubMed between 2018 and 2022. We downloaded triplet data from the Semantic MEDLINE Database (SemMedDB) and matched them with the retrieved literature to obtain triplet data for the target articles. We visualized the triplet information using NetworkX for knowledge discovery.

**Results:**

Within the scope of literature research in the past 5 years, malignant neoplasms appeared most frequently (587/1387, 42.3%). Pharmacotherapy (267/1387, 19.3%) was the primary treatment method, with trastuzumab (209/1805, 11.6%) being the most commonly used therapeutic drug. Through the analysis of the knowledge graph, we have discovered a complex network of relationships between treatment methods, therapeutic drugs, and preventive measures for different types of breast cancer.

**Conclusions:**

This study constructed a knowledge graph for breast cancer prevention and treatment, which enabled the integration and knowledge discovery of relevant literature in the past 5 years. Researchers can gain insights into treatment methods, drugs, preventive knowledge regarding adverse reactions to treatment, and the associations between different knowledge domains from the graph.

## Introduction

Breast cancer is the most common malignant tumor in women worldwide, with a reported death toll exceeding 600,000 in 2018 alone [1]. Breast cancer has emerged as the most prevalent cancer and a primary cause of mortality among women. The global incidence of new cases of female breast cancer witnessed a sharp increase from 1.05 million in 2000 to 2.09 million in 2018 [2]. In 2020, global cancer burden data revealed that new breast cancer cases reached 2.26 million, constituting 11.7% of all newly diagnosed cancer cases worldwide. The newly reported mortality cases numbered 0.68 million, representing 6.9% of global newly reported deaths [3]. Factors such as old age, young age at menarche, family history of breast cancer, smoking, and drinking alcohol increase the risk of breast cancer [4-6]. On the contrary, regular physical exercise; breastfeeding; regular work and rest; and intake of fruits, vegetables, whole grains, and dietary fiber can appropriately reduce the risk of breast cancer [7]. Various treatment methods are used for patients with breast cancer, including surgery, radiation therapy, endocrine therapy, chemotherapy, and targeted therapy. So far, most countries have primarily focused on population education for breast cancer prevention, including encouraging increased physical activity, controlling BMI, and limiting alcohol intake [8]. Despite the increasing number of research literature, a large amount of literature on breast cancer prevention and treatment has not been systematically integrated. Knowledge graph technology allows for the independent connection and integration of disparate literature, resulting in a more comprehensive and cohesive knowledge framework.

Knowledge Graph is a knowledge repository proposed by Google in 2012 to enhance the functionality of search engines. It describes concepts and their relationships in the real world using triplets in the form of entity-relation-entity [9]. Knowledge graphs can integrate information from diverse sources and domains, including text, databases, and web pages, and intricately interlink them. These integrations serve to mitigate information silos, fostering the establishment of a more comprehensive knowledge framework. Knowledge graphs have been widely used in various fields, such as medicine, network security, journalism, finance, and education [10]. Knowledge graphs in the biomedical domain have applications in studies related to disease associations [11], genomics [12], drug interactions [13], and support for physicians in formulating individualized treatment regimens [14]. At present, there are well-established knowledge graphs, including DisGeNET [15], which integrate information on the associations between genes and diseases; DrugBank [16], a comprehensive bioinformatics and cheminformatics knowledge base; and ClinVar [17], a compilation of genetic variation information from diverse laboratories worldwide. One study extracted breast cancer–related features from Chinese breast cancer mammography reports and built a knowledge graph for diagnosing breast cancer by combining diagnosis and treatment guidelines and insights from clinical experts [18]. Another study integrated triples from clinical guidelines, medical encyclopedias, and electronic medical records to build a breast cancer knowledge graph [19]. Despite a small number of scholars having constructed knowledge graphs for breast cancer, the varied emphases and diverse data sources employed render their applicability limited. A knowledge graph specifically focused on the prevention and treatment of breast cancer has not been constructed at present. Therefore, this study primarily collects information related to the prevention and treatment of breast cancer to construct a knowledge graph.

In the biomedical field, there are already mature tools (eg, SemRep) for extracting knowledge from medical texts. SemRep is a natural language processing program based on the Unified Medical Language System (UMLS), which performs operations such as text tokenization, syntactic analysis, part-of-speech disambiguation, phrase mapping, semantic predicate normalization, and syntactic constraints [20]. It extracts entities and relationships from biomedical texts and outputs triplets stored in the Semantic MEDLINE Database (SemMedDB) [21]. SemMedDB currently encompasses details on approximately 96.3 million predications derived from all PubMed citations (around 29.1 million citations) and serves as the foundation for the Semantic MEDLINE application [22]. We downloaded the entity and relationship data provided by SemMedDB. NetworkX is an open-source library for Python, primarily designed for creating, analyzing, and visualizing complex network structures. NetworkX plays a significant role in knowledge visualization, facilitating users in intuitively presenting and comprehending intricate knowledge graphs or network data.

## Methods

### Ethics Approval

This study was approved by the Board of Medical Ethics Committee of Shenzhen Maternal and Child Health Hospital (SFYLS[2022]003).

### Data Source

We conducted a search on PubMed using Medical Subject Headings terms “breast cancer,” “prevention,” and “treatment,” covering the period from January 1, 2018, to December 31, 2022, and the study type was clinical trials. A total of 3589 articles were retrieved. We obtained the entity and relationship data from SemMedDB.

### Data Processing and Construction of Knowledge Graph

We matched the PMIDs of the retrieved articles with the database and extracted the corresponding triplet information. We initially obtained 33,060 Subject-Predicate-Object (SPO) triplets of data.

Next, we made improvements according to the SPO cleaning principles proposed by Fiszman et al [9] (ie, relevance, connectivity, novelty, and significance). We combined them with expert manual screening to ensure that the selected SPO triplets have a higher relevance. In the improved process, we did not predefine semantic patterns. Instead, we used a series of cleaning operations to select core SPO triplets and connected SPO triplets, eliminating SPO triplets lacking specific information and those that appeared only once in the frequency. The specific process is as follows:

1. In the same article, there may be repeated occurrences of identical SPO triplets. To maintain equal contribution from each article, we counted the repeated SPO triplets once within the same article.
2. To ensure statistical reliability, we calculated the occurrence frequency of each SPO triplet across different articles. SPO triplets with low occurrence frequencies may lack statistical significance. Therefore, we filtered SPO triplets with frequencies greater than or equal to 2.
3. Based on expert domain knowledge, we manually screened the selected SPO triplets with frequencies greater than or equal to 2 to identify those of research value.

Finally, we obtained 25,449 SPO triplets data. We imported the filtered SPO triplets information into the NetworkX for visual analysis to explore knowledge and information related to breast cancer prevention and treatment.

All analyses were conducted in a Python program (version 3.11.3; Python Software Foundation), primarily using Pandas, Matplotlib, WordCloud, and NetworkX packages [23-26].

## Results

### Summary of Included Literatures

A total of 3589 articles were published in 618 different journals. Among them, 191 articles were published in the same journal, while 293 journals had only 1 article published. The journals were ranked based on the number of publications, and the top 100 journals accounted for 2631 articles, which is 73.30% of the total.

### Semantic Relationships and Semantic Patterns

We mainly summarize semantic associations into 3 types: treatment and prevention, influencing or associated factors, and related diseases (Table S1 in Multimedia Appendix 1). Regarding treatment and prevention, the relationships include TREATS, ADMINISTERED_TO, USES, and PREVENTS, representing treatment drugs, surgeries, and preventive measures for breast cancer. Regarding influencing or associated factors, the relationships include ASSOCIATED_WITH, AFFECTS, and CAUSES, which represent diseases’ impact and etiological factors. Regarding related diseases, the relationship COEXISTS_WITH represents the coexistence between different diseases. In the semantic patterns involving treatment (TREATS), the topp-TREATS-neop and topp-TREATS-podg have appeared over 1000 times.

### Summary of SPO Triples

In terms of breast tumors, malignant neoplasms had the highest frequency, accounting for 42.3% (587/1387) of the total, followed by triple-negative breast neoplasms (56/1387, 4%) and human epidermal growth factor receptor 2 (*HER*2)–positive carcinoma of breast (54/1387, 4%; Table 1 and Multimedia Appendix 2).

**Table 1.** Summary of breast cancer subtypes and stages, treatment methods, and treatment drugs. The top 30 subtypes, treatment methods, and treatment drugs with higher frequencies in all data are presented for each group.

Group		Values, n (%)
**Breast cancer subtypes and stages (n=1387)**		
	Malignant neoplasm of breast	587 (42.3)
	Triple-negative breast neoplasms	56 (4)
	*HER*2^a^-positive carcinoma of breast	54 (3.9)
	Carcinoma breast stage IV	48 (3.5)
	Breast cancer metastatic	47 (3.4)
	Early-stage breast carcinoma	42 (3)
	Malignant neoplasms	31 (2.2)
	Neoplasm	30 (2.2)
	Metastatic triple-negative breast carcinoma	26 (1.9)
	High-risk cancer	24 (1.7)
	Neoplasm metastasis	21 (1.5)
	Advanced cancer	19 (1.4)
	Advanced breast carcinoma	19 (1.4)
	*HER*2-negative breast cancer	18 (1.3)
	Locally advanced malignant neoplasm	17 (1.2)
	Advanced malignant neoplasm	15 (1.1)
	Nonsmall cell lung carcinoma	15 (1.1)
	Noninfiltrating intraductal carcinoma	14 (1)
	Locally advanced breast cancer	13 (0.9)
	Breast cancer stage III	11 (0.8)
**Treatment of breast cancer (n=1387)**		
	Pharmacotherapy	267 (19.3)
	Neoadjuvant therapy	88 (6.3)
	Hormone therapy	68 (4.9)
	Chemotherapy (adjuvant)	54 (3.9)
	Therapeutic procedure	53 (3.8)
	Radiation therapy	48 (3.5)
	Treatment protocols	43 (3.1)
	Adjuvant therapy	36 (2.6)
	Breast-conserving surgery	35 (2.5)
	First-line treatment	31 (2.2)
	Single-agent therapy	27 (1.9)
	Mastectomy	27 (1.9)
	Operative surgical procedures	20 (1.4)
	Interventional procedure	16 (1.2)
	Radiotherapy (adjuvant)	14 (1)
	Excision of axillary lymph nodes group	13 (0.9)
	Combined modality therapy	12 (0.9)
	Excision	11 (0.8)
	Targeted therapy	11 (0.8)
	Placebos	10 (0.7)
**Drugs for breast cancer (n=1805)**		
	Trastuzumab	209 (11.6)
	Capecitabine	88 (4.9)
	Paclitaxel	81 (4.5)
	Aromatase inhibitors	64 (3.5)
	Immunologic adjuvants	62 (3.4)
	Letrozole	58 (3.2)
	Bevacizumab	48 (2.7)
	Tamoxifen	40 (2.2)
	Gemcitabine	36 (2)
	Pertuzumab	36 (2)
	Fulvestrant	36 (2)
	Cyclophosphamide	32 (1.8)
	Pembrolizumab	30 (1.7)
	Docetaxel	27 (1.5)
	Taxane	27 (1.5)
	Ado-trastuzumab emtansine	22 (1.2)
	130-nm albumin-bound paclitaxel	22 (1.2)
	Carboplatin	22 (1.2)
	Eribulin	21 (1.2)
	Palbociclib	19 (1.1)
	Exemestane	19 (1.1)
	Everolimus	19 (1.1)
	Olaparib	18 (1)
	Talazoparib	17 (0.9)
	Pharmaceutical preparations	16 (0.9)
	Protein-tyrosine kinase inhibitor	15 (0.8)
	Cisplatin	14 (0.8)
	Lapatinib	14 (0.8)
	Fluorouracil	13 (0.7)
	Preservative free ingredient	13 (0.7)

a *HER*2: human epidermal growth factor receptor 2.

Pharmacotherapy is the most common treatment method, accounting for 19.2% (267/1387) of the overall frequency. Additionally, other high-frequency treatment modalities include neoadjuvant therapy (88/1387, 6%), hormone therapy (68/1387, 5%), adjuvant chemotherapy (54/1387, 4%), and radiation therapy (48/1387, 3%; Table 1 and Multimedia Appendix 3). In breast cancer treatment drugs, trastuzumab (209/1805, 11.6%), capecitabine (88/1805, 5%), paclitaxel (81/1805, 4%), aromatase inhibitors (64/1805, 4%), and immunologic adjuvants (62/1805, 3%) have a relatively high frequency of occurrence (Table 1 and Multimedia Appendix 4).

### Breast Cancer Knowledge Graph

**Figure 1.**
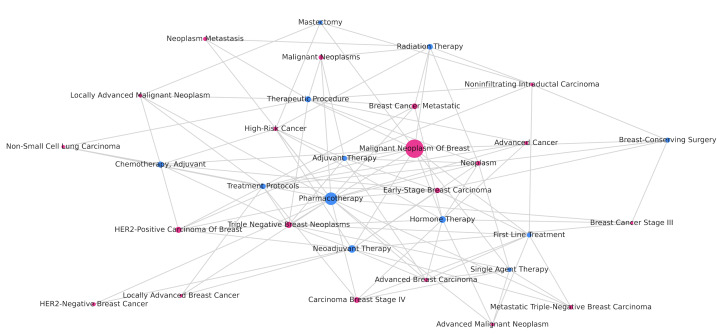
Relationship between different subtypes and stages of breast cancer and treatment methods. *HER*2: human epidermal growth factor receptor 2.

We visualized the SPO triples and displayed 3 subgroups: breast cancer treatment methods, therapeutic drugs, and relevant preventive measures. Figure 1 shows the relationship between different subtypes and stages of breast cancer and treatment methods. In different subtypes of breast cancer, the highest frequency is observed in malignant neoplasm of the breast, with pharmacotherapy having the highest frequency among various treatment modalities. Different subtypes simultaneously correspond to multiple treatment modalities; likewise, a single treatment modality corresponds to multiple breast cancer subtypes.

**Figure 2.**
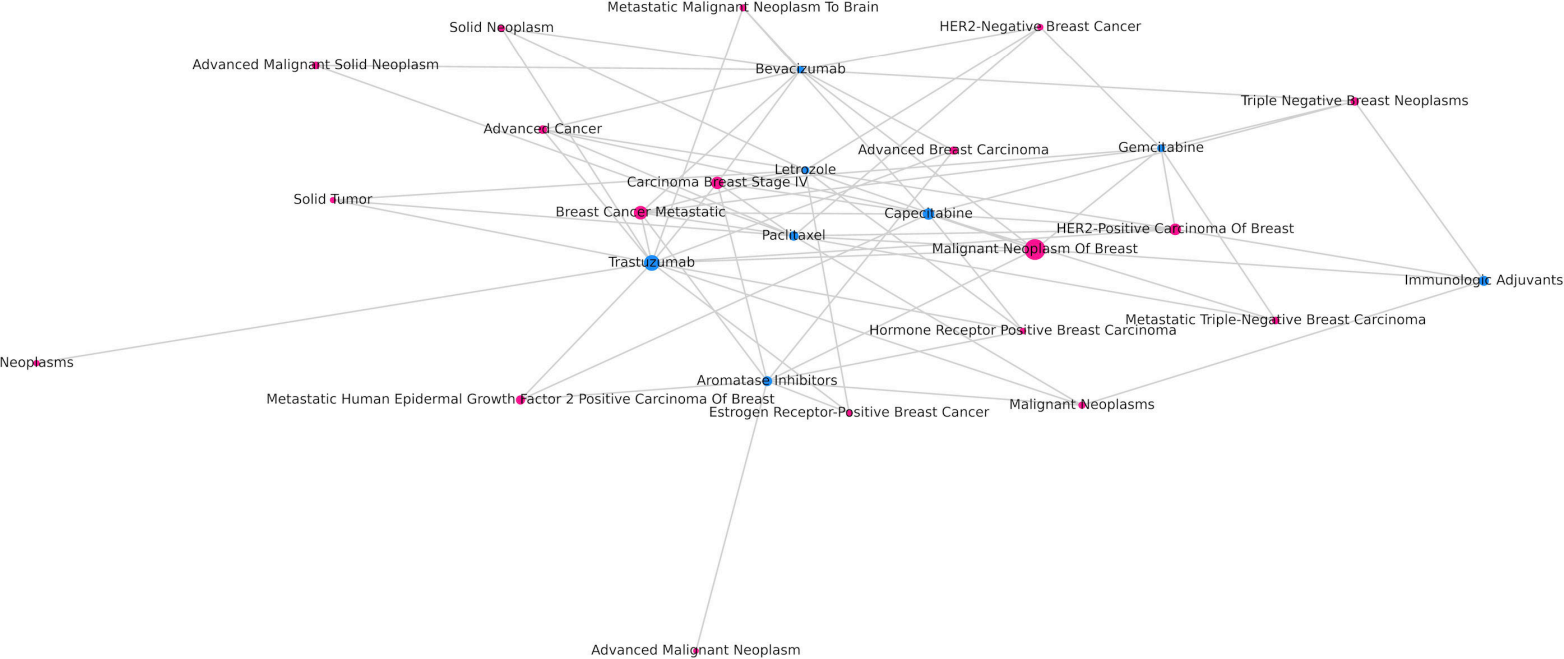
Relationship between different subtypes and stages of breast cancer and drugs. *HER*2: human epidermal growth factor receptor 2.

Figure 2 shows the relationship between different subtypes and stages of breast cancer and drugs. Among the therapeutic drugs for breast cancer, trastuzumab has the highest frequency and corresponds to the most types of breast cancer. Capecitabine, paclitaxel, aromatase inhibitors, and immunologic adjuvants also have relatively high frequencies. In comparison, immunologic adjuvants have the fewest connections with different types of breast cancer.

**Figure 3.**
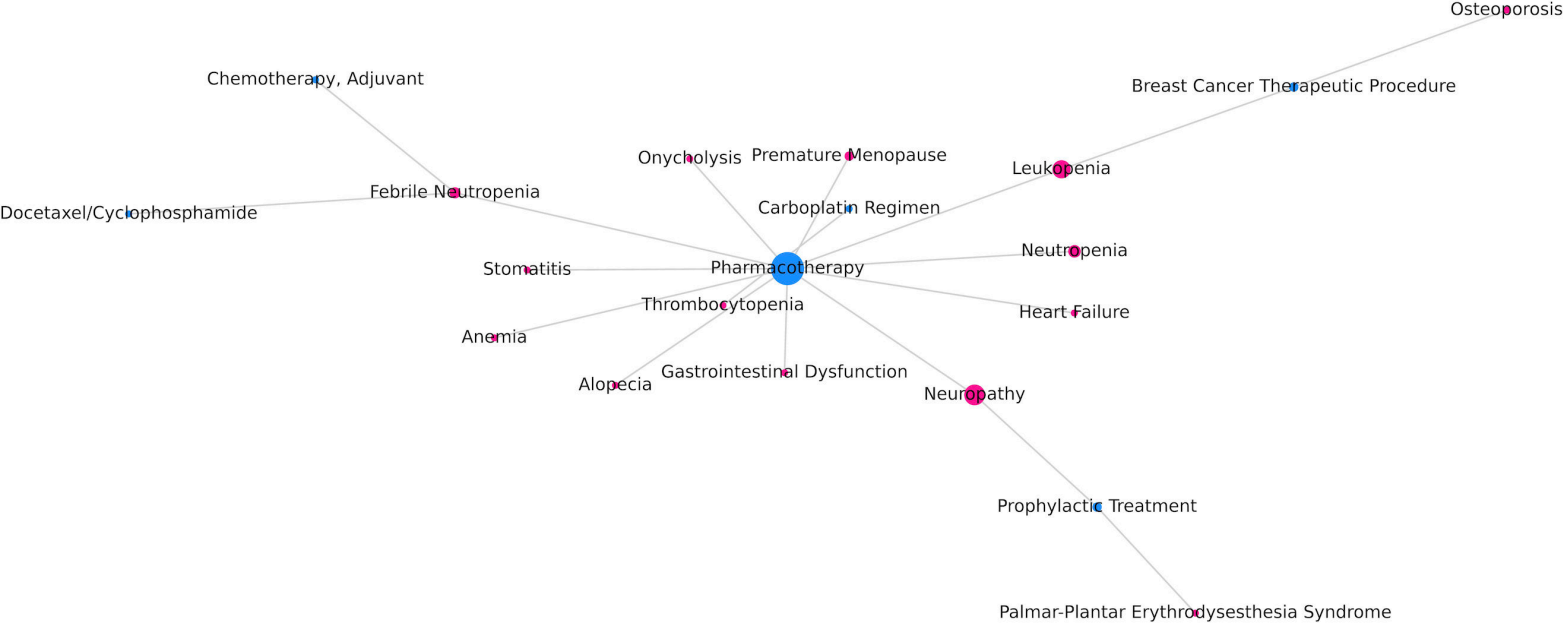
Relationship between breast cancer treatment and adverse reactions.

Figure 3 shows the relationship between breast cancer treatment and adverse reactions. Pharmacotherapy is associated with neuropathy, onycholysis, heart neutropenia failure, alopecia, febrile neutropenia, anemia, stomatitis, leukopenia, thrombocytopenia, premature menopause, and gastrointestinal dysfunction. Additionally, multiple nodes are connected, forming multiple pathways, such as pharmacotherapy-febrile neutropenia-adjuvant chemotherapy and pharmacotherapy-leukopenia-breast cancer therapeutic procedure-osteoporosis.

**Figure 4.**
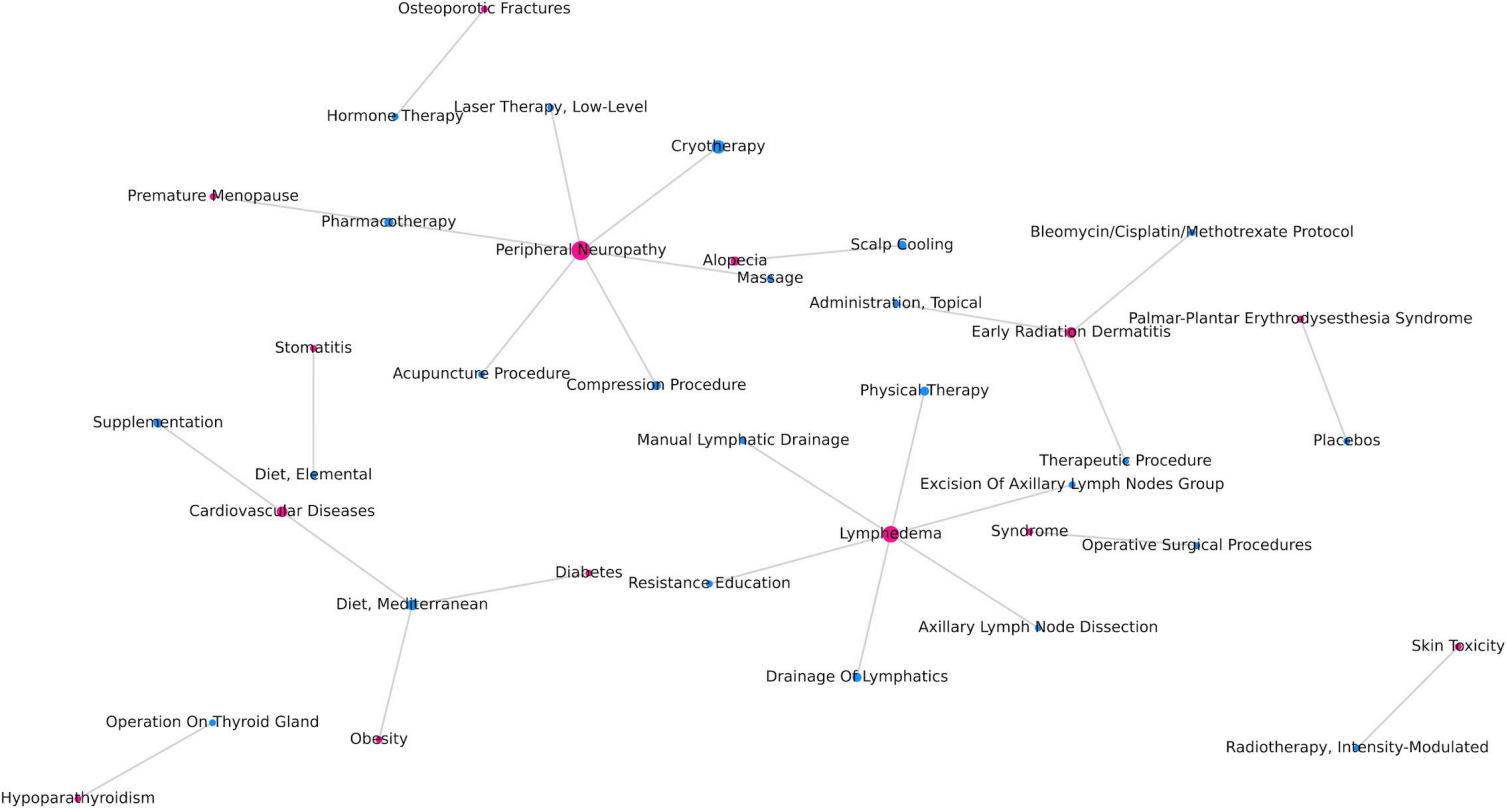
Relationship between adverse reactions after breast cancer treatment and preventive measures.

Figure 4 shows the relationship between adverse events after breast cancer treatment and preventive measures. Peripheral neuropathy is associated with cryotherapy, low-level laser therapy, compression procedure, acupuncture procedure, pharmacotherapy, and massage. Lymphedema is associated with resistance education, axillary lymph node dissection, physical therapy, excision of axillary lymph nodes group, and drainage of lymphatics. Early radiation dermatitis is associated with topical administration and bleomycin, cisplatin, or methotrexate protocol. In addition, there are some adverse reactions with relatively few treatment measures, such as stomatitis-diet, alopecia-scalp cooling.

**Figure 5.**
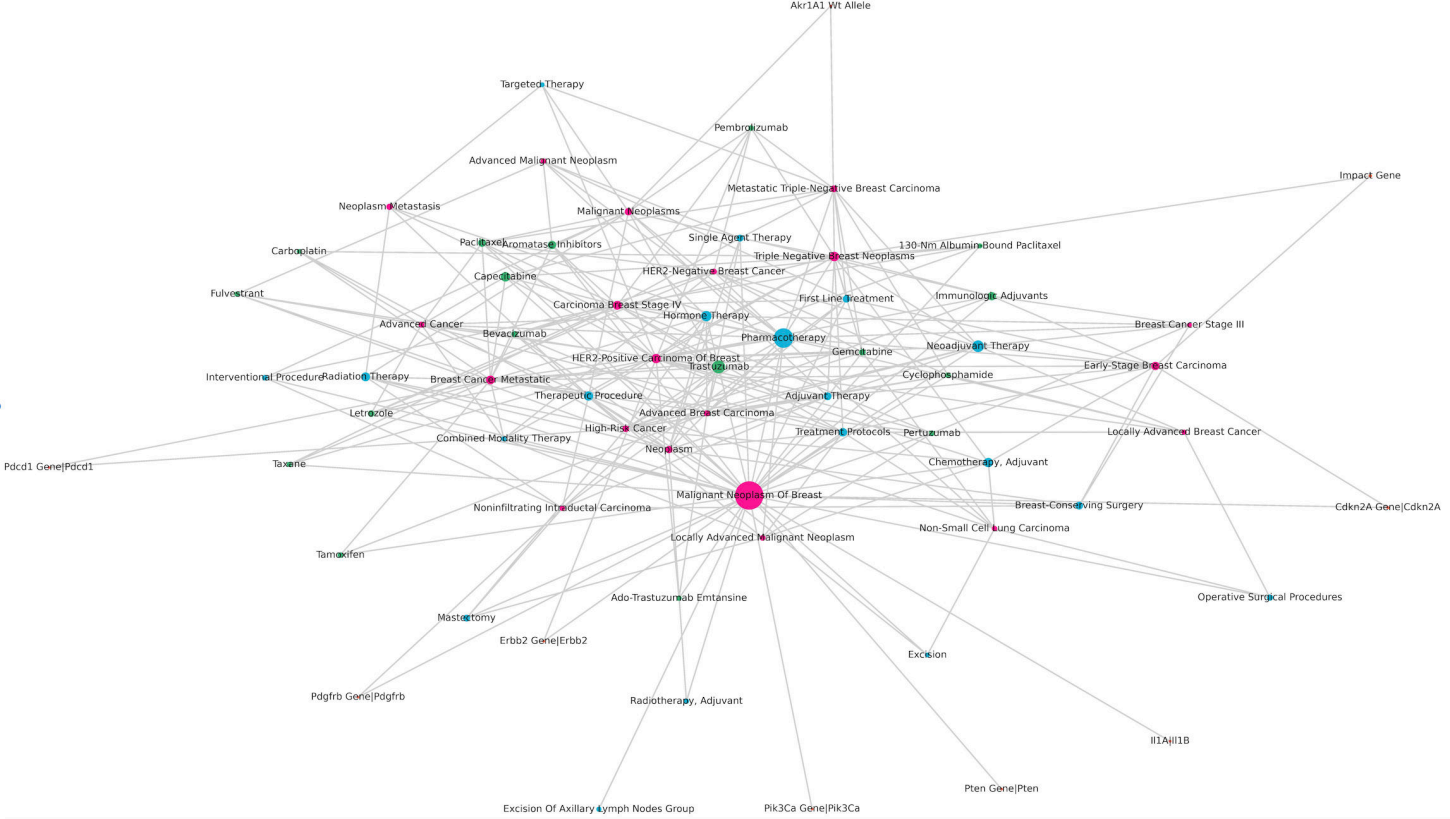
Relationship between types of breast cancer, treatments, drugs, and genes.

We performed a relationship visualization to gain a better understanding of the association between types of breast cancer, treatments, drugs, and genes. Figure 5 intuitively reflects the high frequency of malignant neoplasm of the breast, pharmacotherapy, and trastuzumab. In addition, breast malignant tumors are associated with multiple genes, such as the phosphatidylinositol-4,5-bisphosphate 3-kinase catalytic subunit alpha (*PIK3CA*) gene, platelet-derived growth factor receptor beta (*PDGFRB*) gene, phosphatase and tensin homolog (*PTEN*) gene, and erb-B2 receptor tyrosine kinase 2 (*ERBB2*) gene.

## Discussion

### Principal Findings

The knowledge graphs constructed in this study help researchers understand the research hot spots in breast cancer over the past 5 years. The complex network involving treatment methods, drugs, adverse reactions, preventive measures, and genes in breast cancer can assist clinicians in making decisions that comprehensively consider multiple aspects, ultimately aiding in decisions that are the most beneficial to patients. Additionally, the knowledge graph allows for personalized considerations based on specific genes for individualized patients.

This study found that from 2018 to 2022, breast malignancies appeared most frequently in the literature and were the primary concern for researchers. Research interest in triple-negative breast neoplasms is higher than in other subtypes. This phenomenon may be due to the higher risk of recurrence and poor prognosis in patients with early-stage triple-negative breast neoplasms [10], making it a subject of greater concern to clinicians and researchers. Among treatment modalities, pharmacotherapy receives the highest attention. Pharmacotherapy for breast cancer primarily involves chemotherapy, endocrine therapy, and targeted therapy [27]. Compared to traditional surgery and radiotherapy, pharmacotherapy can more precisely intervene in the growth and division of cancer cells by targeting specific molecules or cellular structures, which reduces damage to normal cells and allows for the formulation of personalized treatment plans based on the patient’s genotype and molecular characteristics [28]. Medications circulating through the bloodstream can also act on cancer cells throughout the body, preventing cancer cell metastasis. These advantages of pharmacotherapy may be related to the heightened emphasis on pharmacotherapy over the past 5 years. Trastuzumab receives the highest attention in breast cancer pharmacotherapy; it is a specific cancer-targeting medication used in the treatment of cancers characterized by elevated levels of HER2 protein [29].

Pharmacotherapy is associated with various adverse reactions, including neutropenia, neuropathy, onycholysis, heart failure, alopecia, and febrile neutropenia. Among these adverse reactions, peripheral neuropathy and lymphedema have the most corresponding preventive and treatment measures, with lymphedema being a common complication after surgery [30]. However, there is limited research on how to prevent and treat the potential adverse reactions of pharmacotherapy, and further studies are needed. Various adverse effects of breast cancer treatment may reduce patients’ adherence to treatment. Therefore, when clinicians choose different treatments and drugs, they should pay close attention to their potential adverse reactions and how to prevent or mitigate them.

In existing knowledge graphs related to breast cancer, one study from China constructed a knowledge graph using electronic medical records, clinical guidelines, and expert opinions, primarily focusing on breast cancer diagnosis [18]. Another study by Chinese scholars also used data from various sources, including clinical guidelines, medical encyclopedias, and electronic medical records, to construct a knowledge graph primarily applied to medical knowledge question-answering and medical record retrieval [19]. These studies used data from multiple sources, including structured, unstructured, and semistructured data. Data extraction and accuracy face challenges. Therefore, they used neural network models for training and calculated a series of metrics to ensure data accuracy. For instance, they utilized BERT + Bi-LSTM+ CRF for textual data to achieve named entity recognition. In this study, SemMedDB was used as the data source, and the database was constructed by extracting semantic information from PubMed using SemRep, which demonstrated good performance in a biomedical text [21].

In summary, the knowledge graph constructed in this study for breast cancer treatment and prevention encompasses information on different stages, subtypes of breast cancer, treatment modalities, medications, adverse reactions, and preventive measures. This knowledge forms a complex network, providing clinical practitioners with a comprehensive and referenced knowledge base. We recommend that clinical practitioners apply our research findings in several aspects. First, clinicians can gain insights into the current state of breast cancer treatment and prevention research through our study. Additionally, there is a relative lack of preventive measures and strategies for mitigating postoperative and postmedication adverse reactions compared to breast cancer treatment, and more efforts are needed in these areas. Furthermore, our research can assist clinicians in making comprehensive decisions. For instance, when selecting a treatment approach for patients, the knowledge graph facilitates linking to available medications, associated adverse reactions, and measures to mitigate or prevent adverse effects.

Our research still has several limitations. First, SemRep, as a natural language processing program based on the UMLS, still exhibits shortcomings. Despite the extensive coverage and scale of the UMLS Metathesaurus, it has a relatively limited ability to recognize entities. There are still areas for improvement in processing natural language texts [20]. Second, clinical researchers often prefer causal relationships rather than pure correlations; however, our study can only reveal the connections between pieces of information and cannot determine the magnitude and direction of their effects. Third, with the release of new literature, the knowledge graph also needs to be updated promptly, increasing the burden on researchers. Future improvements should focus on automating the mining of literature data to ensure timely updates to the knowledge graph for breast cancer prevention and treatment, thereby alleviating the burden on researchers.

### Conclusions

This study successfully constructed a knowledge graph for breast cancer prevention and treatment by integrating relevant literature from the past 5 years and conducting knowledge discovery. Through this knowledge graph, researchers can learn about breast cancer treatment methods, medications, and adverse reactions to preventive treatments and gain insights into the relationships between different pieces of knowledge.

## Supplementary material

10.2196/52210 Multimedia Appendix 1 Table depicting the semantic relationship and semantic schema of breast cancer.

10.2196/52210 Multimedia Appendix 2 Different subtypes and stages of breast cancer.

10.2196/52210 Multimedia Appendix 3 Treatments of breast cancer.

10.2196/52210 Multimedia Appendix 4 Drugs for breast cancer.

## Abbreviations

ERBB2: erb-B2 receptor tyrosine kinase 2
HER2: human epidermal growth factor receptor 2
PDGFRB: platelet-derived growth factor receptor beta
PIK3CA: phosphatidylinositol-4,5-bisphosphate 3-kinase catalytic subunit alpha
PTEN: phosphatase and tensin homolog
SemMedDB: Semantic MEDLINE Database
SPO: Subject-Predicate-Object
UMLS: Unified Medical Language System
