# Supplementary material for: Knowledge Graph for Breast Cancer Prevention and Treatment: Literature-Based Data Analysis Study
Source: JMIR Med Inform. 2024 Feb 22;12:e52210. doi: 10.2196/52210 (PMC11004512; doi:10.2196/52210)
Supplement: Multimedia Appendix 1 [file medinform-v12-e52210-s001.docx]

Table S1. Semantic relationship and semantic schema of breast cancer.

| Type | Predicate | Semantic Schema Example | Frequency | SPO Example |
| --- | --- | --- | --- | --- |
| **Treatment** | | | | |
|  | TREATS | topp - TREATS - neop | 1387 | Radiation therapy TREATS Malignant neoplasm of breast |
|  |  | topp - TREATS - podg | 1128 | Radiation therapy TREATS Patients |
|  |  | phsu - TREATS - neop | 909 | Pegfilgrastim TREATS Malignant neoplasm of breast |
|  |  | phsu - TREATS - podg | 896 | Acetaminophen TREATS Patients |
|  |  | orch - TREATS - neop | 346 | Tamoxifen TREATS Malignant neoplasm of breast |
|  |  | topp - TREATS - popg | 298 | Chemotherapy, Adjuvant TREATS Woman |
|  |  | phsu - TREATS - popg | 209 | Paclitaxel TREATS Population Group |
|  | ADMINISTERED_TO | topp - ADMINISTERED_TO - humn | 614 | Pharmacotherapy ADMINISTERED_TO Patients |
|  |  | orch - ADMINISTERED_TO - humn | 323 | Epirubicin ADMINISTERED_TO Patients |
|  | USES | topp - USES - phsu | 479 | Adjuvant therapy USES Aromatase Inhibitors |
|  |  | topp - USES - aapp | 155 | Therapeutic procedure USES Bevacizumab |
| **Prevention** | PREVENTS | phsu-PREVENTS-sosy 41 | 41 | Vitamin B 12 PREVENTS Pain |
|  |  | topp-PREVENTS-dsyn 36 | 36 | Administration, Topical PREVENTS Early radiation dermatitis |
|  |  | topp-PREVENTS-sosy 44 | 44 | Music Therapy PREVENTS Fatigue |
| **Influencing/Related factors** | | | | |
|  | ASSOCIATED_WITH | aapp - ASSOCIATED_WITH - neop | 234 | Trastuzumab ASSOCIATED_WITH Malignant neoplasm of breast |
|  |  | bact - ASSOCIATED_WITH - dsyn | 1 | Akkermansia muciniphila ASSOCIATED_WITH Obesity |
|  | AFFECTS | topp - AFFECTS - ortf | 9 | Exercise, Aerobic AFFECTS Cardiac function |
|  |  | comd - AFFECTS - neop | 6 | Somatic mutation AFFECTS Malignant neoplasm of urinary bladder |
|  | CAUSES | neop - CAUSES - neop | 3 | Melanoma CAUSES Malignant neoplasm of lung |
|  |  | topp - CAUSES - dsyn | 39 | Pharmacotherapy CAUSES Onycholysis |
| **Related diseases** | | | | |
|  | COEXISTS_WITH | fndg-COEXISTS_WITH-dsyn | 24 | Sedentary lifestyle COEXISTS_WITH Disease |
|  | PRECEDES | dsyn - PRECEDES - patf | 1 | Skin toxicity PRECEDES Mucositis |
|  |  | dsyn - PRECEDES - dsyn | 1 | Hypertensive disease PRECEDES Neutropenia |

Abbreviations: aapp-Amino Acid, Peptide, or Protein; bact-Bacterium; bpoc-Body Part, Organ, or Organ Component; comd-Cell or Molecular Dysfunction; dsyn- Disease or Syndrome; humn-Human; neop-Neoplastic Process, orch-Organic Chemical; patf-Pathologic Function; phsu-Pharmacologic Substance; podg- Patient or Disabled Group; popg- Population Group; topp-Therapeutic or Preventive Procedur.
