# Supplementary figures and images for: Knowledge Graph for Breast Cancer Prevention and Treatment: Literature-Based Data Analysis Study
Source: JMIR Med Inform. 2024 Feb 22;12:e52210. doi: 10.2196/52210 (PMC11004512; doi:10.2196/52210)

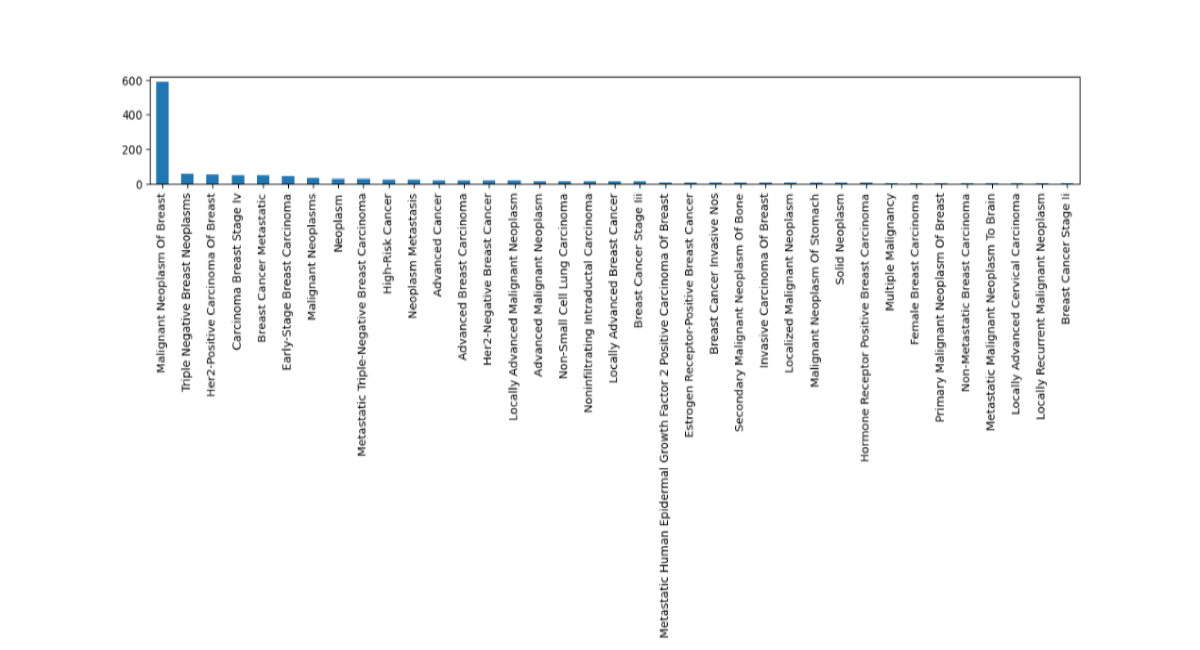

Supplement: Multimedia Appendix 2 [file medinform-v12-e52210-s002.png]

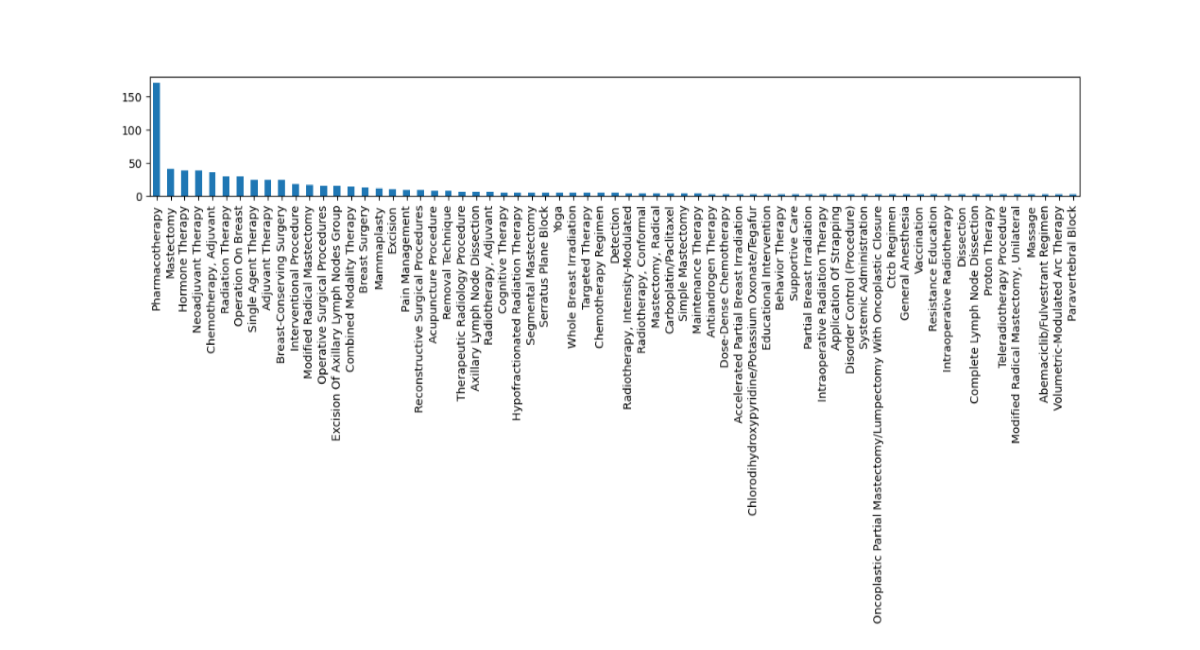

Supplement: Multimedia Appendix 3 [file medinform-v12-e52210-s003.png]

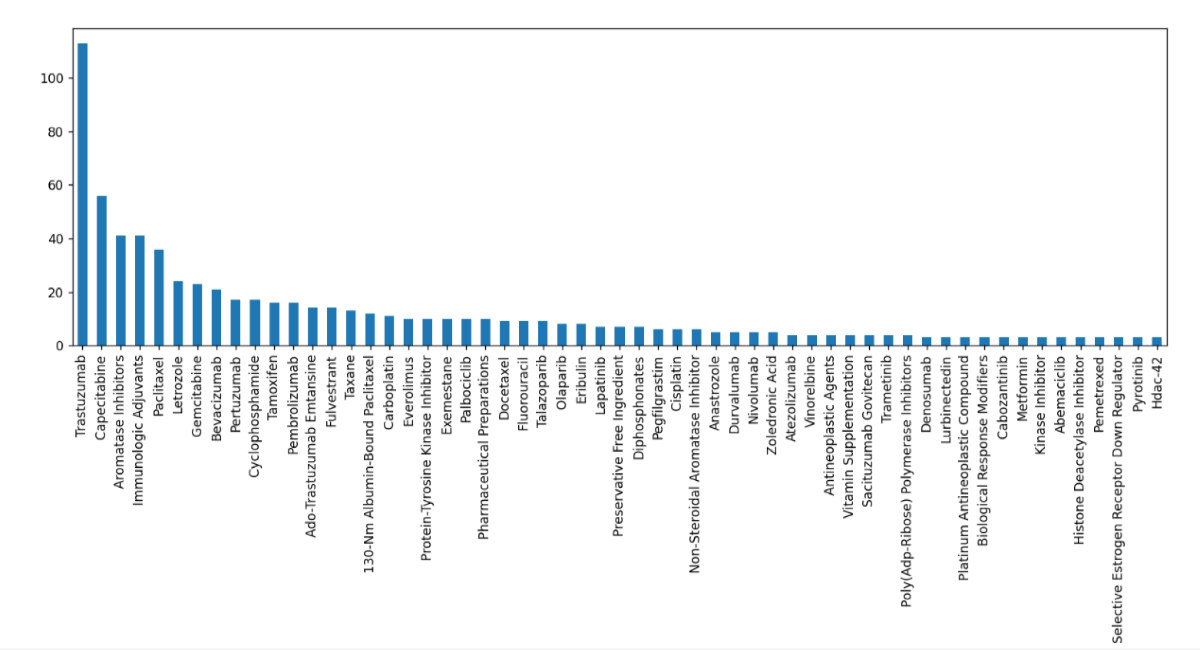

Supplement: Multimedia Appendix 4 [file medinform-v12-e52210-s004.png]
